# Potential anti-EBV effects associated with elevated interleukin-21 levels: a case report

**DOI:** 10.1186/s12879-020-05609-z

**Published:** 2020-11-23

**Authors:** Kristian Assing, Christian Nielsen, Marianne Jakobsen, Charlotte B. Andersen, Kristin Skogstrand, Shahin Gaini, Birgitte Preiss, Sussi Bagge Mortensen, Marianne Nielsine Skov, Line Dahlerup Rasmussen

**Affiliations:** 1 Department of Clinical Immunology, Odense University Hospital, J.B. Winsloevs Vej 4, 5000 Odense, Denmark; 2 Department of Clinical Genetics, Odense University Hospital, Odense, Denmark; 3 Department of Congenital Disorders, Center for Neonatal Screening, Statens Serum Institut, Copenhagen, Denmark; 4 Department of Infectious Diseases, Odense University Hospital, Odense, Denmark; 5 Department of Pathology, Odense University Hospital, Odense, Denmark; 6 Department of Clinical Microbiology, Odense University Hospital, Odense, Denmark

**Keywords:** Case report, Interleukin-21, CD4 T cells, EBV disease, Germinal center, memory B cells, Bcl-6, Peripheral T follicular helper cells

## Abstract

**Background:**

Germinal center derived memory B cells and plasma cells constitute, in health and during EBV reactivation, the largest functional EBV reservoir. Hence, by reducing germinal center derived formation of memory B cells and plasma cells, EBV loads may be reduced. Animal and in-vitro models have shown that IL-21 can support memory B and plasma cell formation and thereby potentially contribute to EBV persistence. However, IL-21 also displays anti-viral effects, as mice models have shown that CD4^+^ T cell produced IL-21 is critical for the differentiation, function and survival of anti-viral CD8^+^ T cells able to contain chronic virus infections.

**Case presentation:**

We present immunological work-up (flow-cytometry, ELISA and genetics) related to a patient suffering from a condition resembling B cell chronic active EBV infection, albeit with moderately elevated EBV copy numbers. No mutations in genes associated with EBV disease, common variable immunodeficiency or pertaining to the IL-21 signaling pathway (including hypermorphic *IL-21* mutations) were found. Increased (> 5-fold increase 7 days post-vaccination) CD4^+^ T cell produced (*p* < 0.01) and extracellular IL-21 levels characterized our patient and coexisted with: CD8^+^ lymphopenia, B lymphopenia, hypogammaglobulinemia, compromised memory B cell differentiation, absent induction of B-cell lymphoma 6 protein (Bcl-6) dependent peripheral follicular helper T cells (pT_FH_, *p* = 0.01), reduced frequencies of peripheral CD4^+^ Bcl-6^+^ T cells (*p* = 0.05), compromised plasmablast differentiation (reduced protein vaccine responses (*p* < 0.001) as well as reduced Treg frequencies. Supporting IL-21 mediated suppression of pT_FH_ formation, pT_FH_ and CD4^+^ IL-21^+^ frequencies were strongly inversely correlated, prior to and after vaccination, in the patient and in controls, Spearman’s rho: − 0.86, *p* < 0.001.

**Conclusions:**

To the best of our knowledge, this is the first report of elevated CD4^+^ IL-21^+^ T cell frequencies in human EBV disease. IL-21 overproduction may, apart from driving T cell mediated anti-EBV responses, disrupt germinal center derived memory B cell and plasma cell formation, and thereby contribute to EBV disease control.

## Background

Epstein-Barr virus (EBV) infects 90–95% of adults worldwide. In health and in EBV lymphoproliferative disorders, memory B cells and plasma cells, collectively, constitute the largest functional EBV reservoir [1, 2]. Interestingly, the increase in EBV loads, associated with immunosuppression, can be accounted for by an increase of latently infected, germinal center (GC) derived, memory B cells [3]. Due to the immunosuppression, a fraction of (circulating) memory B cells can now complete spontaneous lytic replication and thereby increase the EBV load [3]. Interleukin-21 is a GC derived cytokine which may impact EBV persistence in several ways. Interleukin-21 was shown to induce, in an EBV^+^ cell line, which resembled post-GC B cells, increased latent membrane protein 1 (LMP1) and Epstein-Barr virus nuclear antigen (EBNA1) expression, Ig secretion and plasmablast differentiation [4], suggesting that IL-21 may support lytic EBV replication. Mice studies have documented, that interleukin (IL)-21, contributes to the formation of T follicular helper cells and thereby supports GC derived memory B cell and plasma cell differentiation [5]. However, IL-21 has potentially potent anti-EBV effects too. Firstly, animal models have documented, that effector CD8^+^ T cells, capable of killing virus infected cells, require the presence of IL-21 producing CD4^+^ T cells [6]. Secondly, in-vitro studies have shown, that IL-21 is pro-apoptotic for activated and resting B cells [7].

We provide the first documentation that elevated CD4^+^ IL-21^+^ T cell frequencies accompany human EBV disease. Furthermore, our case indicates several anti-EBV effects of sustained and increased IL-21 levels.

## Case presentation

**Fig. 1:**
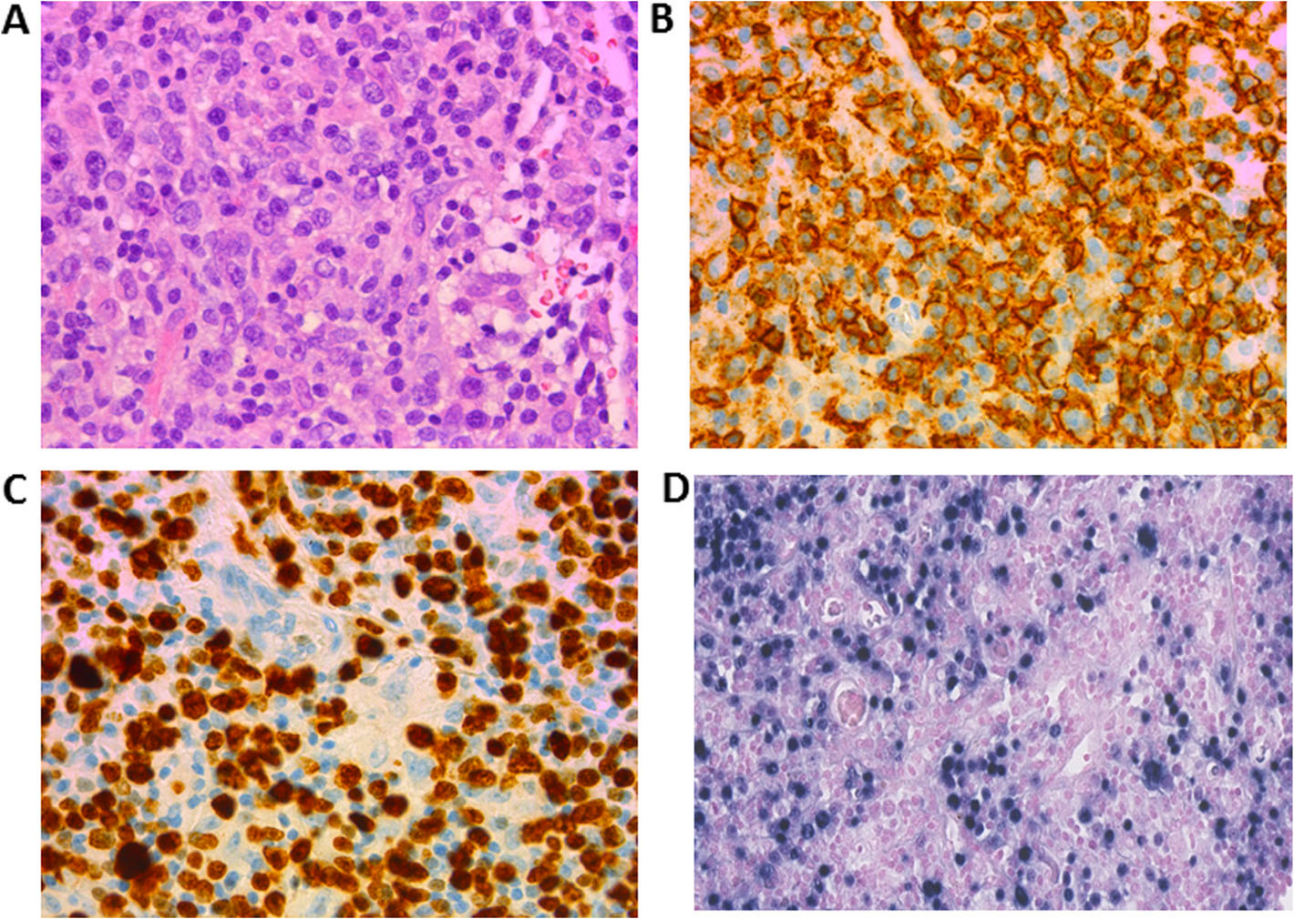
High power fields (40-fold magnification) of the patient’s lung tissue, demonstrating accumulation of: **a** large HE stained cells, **b** positive for CD20^+^ while also displaying extensive coloring for **c** the proliferation marker Ki-67^+^ and for **d** *Epstein–Barr virus*-encoded small RNAs (EBER, Ventana, Tucson, USA)

**Due to undiagnosed progressive pulmonary infiltrates, a 67-year old lady of Danish ancestry, non-smoker, with no infectious history,** was referred to our university clinic. An open lung biopsy revealed a mixed lymphocyte cell infiltrate dominated by CD3^+^ T cells and by areas with centroblastic/ immunoblastic/ Hodgkin-like CD20^+^ B cells. The proliferation marker Ki-67^+^ was extensively expressed (Fig. 1). CD30^+^ immunoblasts were also visible. EBV positive cells (more than 50 per high power field) were found by EBV RNA in-situ hybridization using a commercial (Ventana, Tucson, USA) Epstein-Barr virus small encoded RNA (EBER) probe (Fig. 1). PCR for immunoglobulin heavy-chain gene rearrangements identified a smaller population of B cells with clonal IgH gene-rearrangements placed on a polyclonal background. Clonal rearrangements of the TCR beta-chain were not found. **Lung tissue CD3**^**+**^ **T cells were dominated by CD4**^**+**^ **T cells but perforin and granzyme B positive CD8**^**+**^ **T cells were also present**. Our hematopathologist’s concluding diagnosis was lymphomatoid granulomatosis grade III. The EasySep Human B cell enrichment Kit (STEMCELL™ technologies), which functions optimally with fresh cells, was used on thawed cryopreserved patient and controls PBMC. Among negatively selected patient CD19^+^ B cells (suspension 1) and among the remaining cells (positively selected patient CD4^+^ T cells and CD16^+^/CD56^+^ NK-cells, suspension 2), we determined EBV copy numbers by DNA amplification using the Lightcycler 480 (Roche Diagnostics). DNA, isolated from 200 μL of B cell enriched suspension 1, which contained 46.000 cells (with 53% B cells), generated 13.500 EBV copies, resulting in an average EBV-copy number per cell = 0.29 copies. Similarly, DNA, isolated from 200 μL of B cell depleted suspension 2, which contained 436.000 cells (with 5% B cells), generated 65.900 EBV copies, resulting in an average EBV-copy number per cell = 0.15 copies. Assuming that the average EBV copy number, among the non-B cells in the B cell enriched suspension 1, was also 0.15 copies, suggested that the B cell EBV copy number was even higher than 0.29 copies. In comparison, both suspension 1 and 2, from a healthy IgG anti-VCA positive control, were EBV copy negative.

**Fig. 2:**
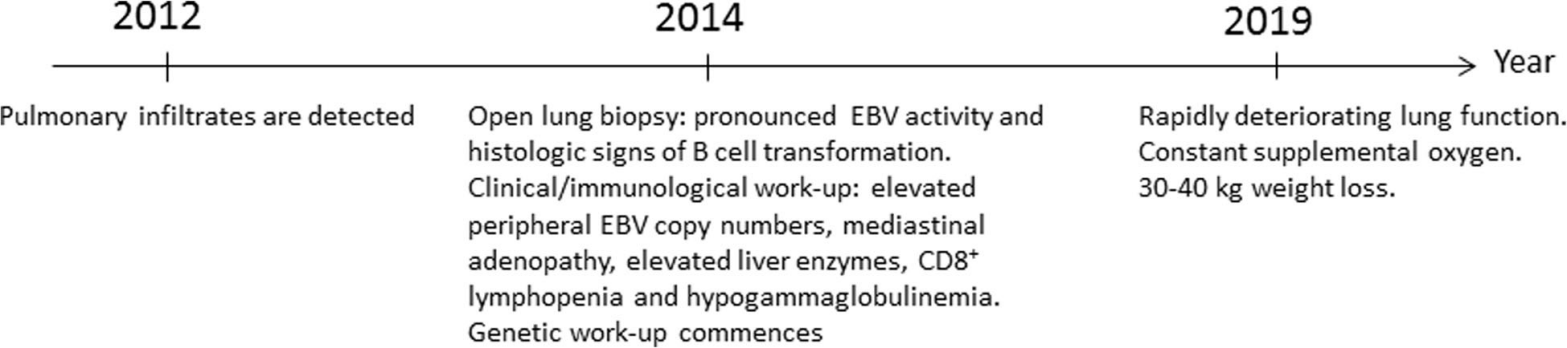
Time line of clinical history. 2012: pulmonary infiltrates are detected. 2014: An open lung biopsy is performed revealing pronounced EBV activity and histologic signs of B cell transformation. Elevated peripheral EBV copy numbers, mediastinal adenopathy, elevated liver enzymes, CD8^+^ lymphopenia and hypogammaglobulinemia are also documented and immunologic and genetic work-up commences. 2019: rapidly deteriorating lung function and 30–40 kg weight loss

**Fig. 3:**
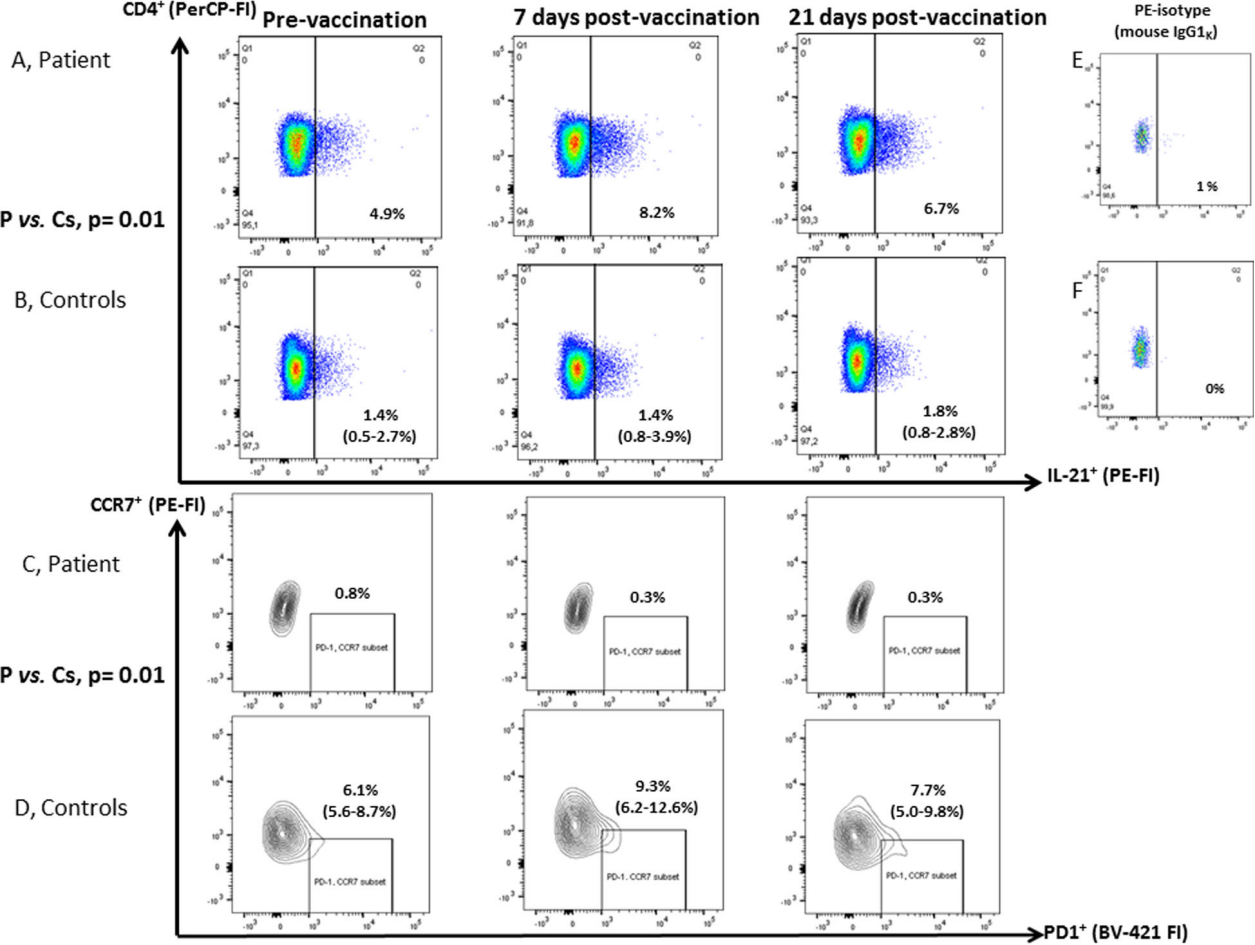
Frequencies of CD4^+^ IL-21^+^ T cells and pT_FH_ prior to and after vaccination. PBMC cultures (1 × 10^6^/mL), sampled pre-, seven and 21 days post-vaccination, were stimulated for three hours with PMA (20 ng/mL) and ionomycin (300 ng/mL). Staining for surface CD4^+^ and intra-cellular IL-21 was performed on PBMC derived from the patient **a** and three (2 of whom were age matched) female controls **b** (patient vs. controls (all time points), *p* = 0.01, Mann- Whitney U test). Frequencies of CD4^+^ CD45RA^−^ CXCR5^+^ CCR7^low^ PD-1^high^ p (eripheral) T_FH_ were ascertained ex-vivo, using the gating strategy devised by He et al. [8], in PBMC (1 × 10^6^/mL), sampled pre-, seven and 21 days post-vaccination. Frequencies of CCR7^low^ and PD-1^high^ expression among patient **c** and controls **d** CD4^+^ CD45RA^−^ CXCR5^+^ T cells are shown (patient vs. controls (all time points), *p* = 0.01, Mann-Whitney U test). Median and minimum-maximum values are shown. The dot-plots for the PE-conjugated isotype control (mouse IgG1_K_) antibody in PMA and inonomycin stimulated CD4^+^T cells from **e** the patient and **f** a control. FI: fluorescence intensity, PE: phycoerythrin, BV: Brilliant Violet, PerCP: Peridinin-Chlorophyll-Protein

**Fig. 4:**
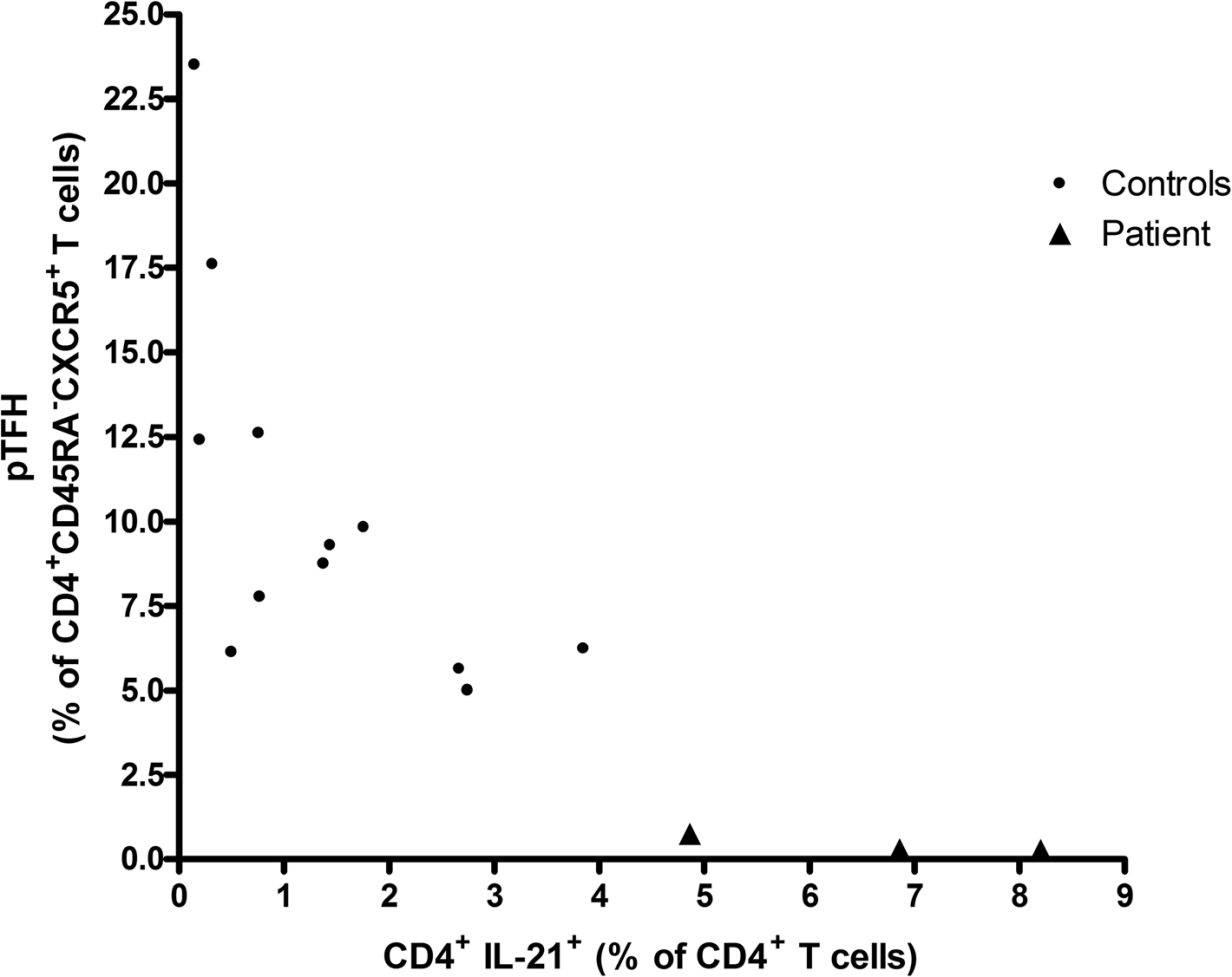
Correlation between frequencies of pT_FH_ and CD4^+^ IL-21^+^ T cells. The inverse correlation (Spearman’s rho: − 0.86, *p* < 0.001) between frequencies of CCR7^low^ and PD-1^high^ pT_FH_ (among CD4^+^ CD45RA^−^ CXCR5^+^ T cells) and CD4^+^ IL-21^+^ T cells, collected prior to, seven and 21 days after vaccination (*n* = 5 subjects and 15 time points). The three time points for the patient are depicted as triangles

A (positron emission tomography) PET scan revealed mediastinal adenopathy with increased fluorodeoxyglucose (FDG) uptake. The hematologists found no indication for chemotherapy. The patient had circulating IgG, but no IgM, specific for (EBNA, concentration: 47.5 U/mL, cut-off: > 20 U/mL) and viral capsid antigen (VCA, concentration: 577 U/mL, cut-off: 20 U/mL). EBV plasma DNA levels were repeatedly (5x over a period of three years) moderately elevated (from < 1000 copies/ml (positive) to 3905 copies/ml). By serology and PCR, she tested negative for human immunodeficiency virus, hepatitis B and C virus, cytomegalovirus, parvovirus, varicella zoster virus, and syphilis. High titer anti-nuclear antibodies (ANA) were present (homogeneous nuclear staining, titer > 1280 (+++), normal range: 0–160) (Table 1). Auto-antibody specificities (anti- Cyclic Citrullinated Peptide (CCP), IgM rheumatoid factor, SS-A Ro52, SS-A Ro60, SS-B, Sm, ribo-nuclear protein (RNP), Scl-70 and Jo-1, Table 1) were negative. Markers for auto-immune hepatitis (soluble liver antigen (SLA) antibodies, liver cytosol (LC1) antibodies, antimitochondrial antibodies (AMA), ACTIN, liver-kidney microsomes (LKM) antibodies) were also negative and clinical signs of autoimmunity were absent. Patient 25-Hydroxy –Vitamin D3 levels were repeatedly (69 and 105 nmol/L) normal. A pan human leukocyte antigen (HLA)-I class specific antibody (e-bioscience clone W6/32) demonstrated that the patient’s leukocytes expressed HLA-class I similar to that of a control (data not shown). HLA-genotyping revealed homozygosity for HLA-A*01, 01; HLA-B*08, 08 and HLA-C*07, 07 but not for HLA-DRB1*03, 13 or HLA-DQB1*02, 06. The patient displayed prolonged elevated lactate dehydrogenase (> 255 U/L), alkaline phosphatase (> 105 U/L) and alanine transaminase (> 45 U/L) levels, consistent with hepatitis. Due to markedly reduced IgA, IgG and IgM levels (Table 1), intravenous immunoglobulin substitution was initiated. Lately, the patient has experienced a 30–40 kg weight loss, her pulmonary function is rapidly deteriorating and she constantly relies on supplemental oxygen (Fig. 2). **In agreement with informed written consent and the study protocols (S-20150176 and S-20192000-48), immunologic work-up was initiated.** A peripheral blood count revealed monocytosis (Table 1) and **f**low cytometry revealed reduced CD19^+^ B cell concentrations, reduced frequencies of CD19^+^ CD27^+^ IgD^−^ (GC derived) memory B cells, normal CD4^+^ T cell concentrations but extremely low CD8^+^ T cell concentrations (Table 1). Patient CD3^−^ CD16^+^/CD56^+^ natural killer (NK) cell concentrations (> 130 × 10^6^/L) were normal. Next generation sequencing found no variants in a targeted panel of genes associated with EBV disease: *SH2D1A, PRF1, XIAP, CD27, CTPS1, RASGRP1, CD70, RLTPR, ITK, MAGT1, PRKCD, UNC13D, STX11, STXBP2, FAAP24* and *CORO1A*, with CVID: *ICOS, TNFRSF13B (TACI), TNFSF13 (APRIL), TNFRSF13C (BAFF-R),TNFSF12 (TWEAK), CD19, CD81, CR2 (CD21), MS4A1(CD20), LRBA, CTLA4, PLCG2, NFKB1, NFKB2, PIK3CD, PIK3R1, VAV1, RAC2, BLK, IKZF1 (IKAROS) and IRF2BP2* and genes of interest: *BLIMP-1 (PRDM1), BCL-6, CD8A, IL21, IL21R* and *STAT3.* Sanger sequencing of the IL-21 gene promoter revealed no mutations. The tridecavalent pneumococcal conjugate vaccine (PCV) was used to vaccinate two age and gender matched controls and the patient. For all three subjects, this was their first PCV vaccination. Three weeks post-vaccination, our patient’s PCV titers were significantly diminished compared to age and gender matched controls (Table 1) and only the patient failed to generate protective antibody levels to some PCV serotypes: 4, 5, 6B and 18C (data not shown). After vaccination, the patient, in contrast to three controls, responded with a 5-fold increase in CD4^+^ IL-21^+^ frequencies but with no induction of CD4^+^ CD45RA^−^ CXCR5^+^ CCR7^low^ PD-1^high^ (peripheral) T follicular helper cells ((p)T_FH_) (Fig. 3). CD4^+^ IL-21^+^ T cell frequency determination on a patient sample (sampled > 1 year after vaccination) and an additional non-vaccinated control sample, accentuated the difference in peripheral CD4^+^ IL-21^+^ T cell frequencies (patient vs. all controls: Mann-Whitney U -test *p* < 0.01). Contrary to four control sera (2 are shown in Table 1), only patient sera (2 of 4) were positive for IL-21 (30.1 and 37.8 pg/mL). Peripheral T_FH_ and CD4^+^ IL-21^+^ frequencies were negatively correlated among vaccinated subjects (pre-, 7 and 21 days post-vaccination for the patient + 4 controls = 15 time points, Spearman’s rho: − 0.86, *p* < 0.001, Fig. 4). Patient CD4^+^ IL-21^+^ T cells were predominantly CXCR5^−^ (approximately 82%, data not shown). Three days of stimulation with anti-CD3/anti-CD28/IL-2 and subsequent intracellular staining for B-cell lymphoma 6 protein (Bcl-6) revealed that patient peripheral CD4^+^ T cells tended to be less often Bcl-6 positive (*n* = 2 different time points, 31.5%; 26.6–36.6%) than peripheral controls CD4^+^ T cells (*n* = 5 controls, 65.0%; 48.4–83.7%, *p* = 0.05). Among ex-vivo peripheral IL-21^+^ CD4^+^ T cells, only those of the patient were dominated by a chemokine receptor profile (CCR7^−^) consistent with tissue homing (CCR7- / CCR7+ fraction: 1.3 vs. two age and gender matched healthy controls: both 0.8, Table 1). The patient’s CD4^+^ CD25^high^ FoxP3^+^ T_reg_ frequencies were reduced (0.9% of CD4^+^T cells) compared to those of 4 healthy adult controls (median: 2.5%; range: 1.7–3.1% of CD4^+^ T cells). The patient’s healthy son (23 years younger than the patient) had normal CD4^+^ and CD8^+^ T cell concentrations, positive IgG anti-EBNA but no detectable EBV copies in his blood (data not shown).

**Table 1:**
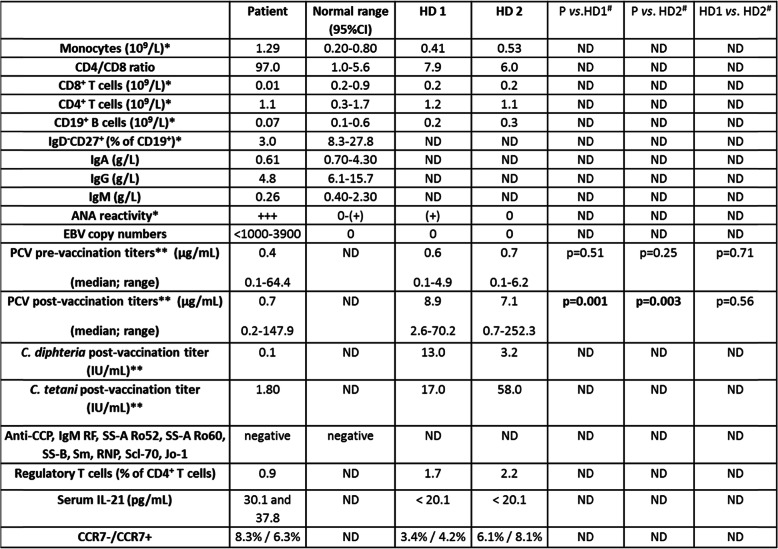
Immunological characteristics associated with chronic EBV reactivation.

*In-house (age adjusted) normal range. ** Protective antibody levels: PCV: 0.35 μg/mL, C. diphteria: > 0.1 IU/mL and C. tetani: > 0.01 IU/mL (Statens Serum Institut, Copenhagen, Denmark), *HD* healthy donor, *ND* not determined. ^#^Mann-Whitney U-test

## Discussion and conclusions

In health and in EBV lymphoproliferative diseases, GC derived memory B cells and plasma cells constitute the primary functional EBV reservoir [2, 3, 9]. Hence, by inhibiting GC derived memory B cell and subsequent plasma cell differentiation, EBV loads may be reduced. We have described the immunological effects associated with an elevated CD4^+^ IL21^+^ profile in an elderly lady suffering from chronic EBV infection where some of the clinical and immunological (severe CD8^+^ lymphopenia, B lymphopenia, hypogammaglobulinemia) findings resembled those of B cell chronic active EBV disease (B cell CAEBV) [10]. However, due to only moderately elevated EBV copy numbers, which is not a typical characteristic of B cell CAEBV [10], we decided to classify her condition, based on the hematopathologist’s diagnosis, as having high grade EBV reactivation. Based on our calculations, we cannot exclude that non-B cells contributed to the patient’s EBV burden. However, according to our calculations, her B cells contained on average at least twice as many EBV-copies than non-B-cells, consistent with the pathological findings. T- and NK- cell tropic EBV infections are rare entities primarily reported among East Asians (our patient was of Danish ancestry). Our histological examination provided no evidence for clonal T cell expansion. T and NK-cell EBV disease also primarily affects children or young adults who develop symptoms as fever, persistent hepatitis, skin symptoms, uveitis, hepatosplenomegaly or pancytopenia [11], none of which characterized our patient. Animal studies have shown, that CD4^+^ T cell produced IL-21 is crucial for anti-viral CD8^+^ T cells [6] and CD8^+^ T cells are critical for controlling EBV [12]. Hence, the elevated CD4^+^ IL-21^+^ T cell frequencies, reported here for the first time in the context of human EBV disease, could be seen as an appropriate response to her severe EBV infection. **We found no mutations in the patient’s** ***IL-21*,** ***IL-21R*, IL-21 promoter or in genes related to EBV disease or CVID. We also excluded auto-immune hepatitis and hepatitis B and C as causes for the elevated patient CD4**^**+**^ **IL-21**^**+**^ **T cell frequencies. Increased IL-21** levels, likely produced by pT_FH,_ have been observed in type I autoimmune hepatitis [13] and chronic hepatitis B [14], however our patient’s **CD4**^**+**^ **IL-21**^**+**^ **T cells were primaly non-**pT_FH_ (CXCR5-)_._ **Collectively, our findings suggest that our patient’s elevated IL-21 profile was indeed secondary to her pulmonary EBV reactivation.** Our patient’s IL-21 producing CD4^+^ T cells were dominated by an effector profile (CCR7^−^) and showed phenotypically no signs of exhaustion (similar PD-1 expression as controls, data not shown). Collectively, this phenotypic profile was consistent with the CD4^+^ IL-21^+^ T cells being able to home to and sustain antiviral CD8^+^ T cell function in peripheral tissues. Our patient’s CD8^+^ lymphopenia is a characteristic also commonly found (44%) among patients with B cell CAEBV [10]. We excluded that her CD8^+^ lymphopenia was secondary to HIV, bare lymphocyte syndrome type I (lack of HLA-class I molecules) [15], vitamin D deficiency [16]*, CD8A* variants or other genetic variants associated with primary immunodeficiency. Furthermore, she had a late onset infectious history and her middle aged son had normal CD8^+^ T cell counts and no EBV reactivation. Collectively, this points to a secondary CD8^+^ T-cell penia, however one fully consistent with the effects of IL-21, since IL-21 causes accumulation of virus specific effector CD8^+^ T-cells in peripheral tissues [17]. Actually, IL-21 induces expression of the gut homing receptor integrin α_4_β_7_ consistent with the intestinal accumulation of CD8^+^ T cells [17]. Accumulation of CD8^+^ T cells in lymph nodes and spleen is also observed in IL-21 transgenic mice [18]. Our patient’s modest EBV copy number elevations also indicated some preserved CD4^+^ and CD8^+^ T-cell functionality in her peripheral tissue. Despite the increased intra- and extracellular IL-21 levels (which likely explained her monocytosis), the patient generated neither pT_FH_ nor protective protein specific antibodies, notwithstanding the central importance of IL-21 for both (p) T_FH_ differentiation [19] and GC derived antibody formation [5]. T follicular helper cells (T_FH_) are located in the GC and are critical for the formation of GC derived memory B cells [20] and plasma cells [21]. As a proxy marker for GC located T_FH_, we focused on their peripheral counterparts: (CD4^+^ CD45RA^−^ CXCR5^+^ CCR7^lo^ PD-1^hi^) pT_FH_ since there are functional and developmental connections between T_FH_ and pT_FH,_ implying that peripheral expansion of pT_FH,_ one week post-vaccination, correlates with GC derived antibody formation [8]. Furthermore, both the differentiation of T_FH_ and pT_FH_ is dependent on the GC transcriptional repressor Bcl-6 [8]. Hence, our patient’s lack of pT_FH_ was consistent with her reduced peripheral CD4^+^ Bcl-6^+^ T cell frequencies.

Priming and differentiation of CD4^+^ IL-21^+^ T cells takes place in lymph nodes, consistent with the increased CD4^+^ IL-21^+^ T cell frequencies, observed seven days post-vaccination (Fig. 3). IL-21 is released within the GC, or in peripheral tissues, and is bound by local IL-21R^+^ cells (B cells, CD4^+^ and CD8^+^ T cells). This might explain why not all patient sera contained elevated IL-21 levels. While having the most pronounced CD4^+^ IL-21^+^ induction among all vaccinees, the patient had absolutely no Bcl-6 dependent pT_FH_ induction day 7. Substantiating a potential antagonism between Bcl-6 dependent pT_FH_ differentiation and the differentiation of CD4^+^IL-21^+^ T cells, we observed a strong inverse correlation between pT_FH_ and CD4^+^ IL-21^+^ formation, pre- and post-vaccination, in patient and controls. This could indicate that other IL-21 producing CD4^+^ T cells subsets [22], apart from pT_FH_ /T_FH_ [8], might either directly compromise the development of the latter or represent alternative GC-derived differentiation pathways. In agreement with this inverse correlation, patient CD4^+^ IL-21^+^ T cells were predominantly CXCR5^−^ and hence of a non-pT_FH_ phenotype. We have not been able to address whether constantly elevated IL-21 levels per se compromised pT_FH_ /T_FH_ formation. Although IL-21 is a Bcl-6 stimulator [5], IL-21 can also suppress intra-nodal Bcl-6 through signal transducer and activator of transcription 3 (STAT3) induced Blimp-1 expression [23]. It can therefore not be excluded, that the patient’s constantly elevated CD4^+^ IL-21^+^ levels could have suppressed intra-nodal, Bcl-6 dependent, pT_FH_ and T_FH_ induction [24] and thereby compromised the patient’s memory B cell formation [20] and subsequent plasmablast generation [21].

Interleukin-21 also induces apoptosis in resting and activated B-cells [7] consistent with the widespread B cell lymphopenia and hypogammaglobulinemia observed in this patient and in a large fraction (42%) of B cell CAEBV patients not treated with rituximab [10]**. Collectively, our data suggests several mechanisms whereby a prolonged increased IL-21 profile might reduce EBV loads: 1) apoptosis of resting and activated B cells combined with compromised GC derived 2) memory B cell and 3) plasma cell differentiation, secondary to disruption of Bcl-6 dependent pT**_**FH**_ **formation.** Due to the very low frequencies of circulating patient memory B cells, the estimation of EBV content in this subset as well as in plasma cells (which are bone marrow resident) is technically not feasible. However, as the B cells constituted the dominant reservoir in our patient, a reduction in memory B cells and plasma cell formation could be a possible mechanism to reduce EBV loads. **In addition,** our patient’s circulating CD4^+^ CD25^high^ FoxP3 T_regs_ frequency was markedly reduced compared to adult controls - a finding potentially attributable to the T_reg_ inhibitory effect of IL-21 [25]. As T_regs_ can inhibit CD8^+^ effector T cell function [26], reduced T_regs_ frequencies could aid CD8^+^ T effector cell efficacy against EBV but could also increase the risk of collateral tissue (lung) damage due to unconstrained CD8^+^ effector T cell activity. The latter might explain her deteriorating lung function. **Consistent with the aforementioned anti-viral mechanisms, the patient had only moderately elevated EBV copy numbers.** We can only speculate, as to why our patient developed high grade EBV reactivation, but her homozygosity for HLA-A* 01, a well-known risk allele for EBV+ tumors [27] could be implicated as well as clonal EBV escape due to extended HLA-class I homozygosity.

We present the first report of elevated CD4^+^ IL-21^+^ T cell frequencies in human EBV disease, thereby complementing animal models documenting that IL-21 is critical for containing chronic viral infections. Secondly, this case suggests that continuous IL-21 overproduction, apart from driving T cell mediated anti-EBV responses, may disrupt GC derived memory B cell and plasma cell formation and thereby diminish the two B cell compartments so important for EBV persistence.

## Data Availability

The datasets used and/or analysed during the current study are available from the corresponding author on reasonable request.

## Abbreviations

IL-21: Interleukin-21
Bcl-6: B-cell lymphoma 6 protein
pT_FH_: Peripheral T follicular helper cells
GC: Germinal center
EBNA1: Epstein-Barr virus Nuclear Antigen 1
LMP1: Latent Membrane Protein 1
B cell CAEBV: B cell chronic active EBV disease
VCA: Viral capsid antigen

## References

[1] Al Tabaa Y, Tuaillon E, Bollore K, Foulongne V, Petitjean G, Seigneurin JM, Duperray C, Desgranges C, Vendrell JP (2009). Functional Epstein-Barr virus reservoir in plasma cells derived from infected peripheral blood memory B cells. Blood.

[2] Calattini S, Sereti I, Scheinberg P, Kimura H, Childs RW, Cohen JI (2010). Detection of EBV genomes in plasmablasts/plasma cells and non-B cells in the blood of most patients with EBV lymphoproliferative disorders by using Immuno-FISH. Blood.

[3] Babcock GJ, Decker LL, Freeman RB, Thorley-Lawson DA (1999). Epstein-barr virus-infected resting memory B cells, not proliferating lymphoblasts, accumulate in the peripheral blood of immunosuppressed patients. J Exp Med.

[4] Konforte D, Simard N, Paige CJ (2008). Interleukin-21 regulates expression of key Epstein-Barr virus oncoproteins, EBNA2 and LMP1, in infected human B cells. Virology.

[5] Linterman MA, Beaton L, Yu D, Ramiscal RR, Srivastava M, Hogan JJ, Verma NK, Smyth MJ, Rigby RJ, Vinuesa CG (2010). IL-21 acts directly on B cells to regulate Bcl-6 expression and germinal center responses. J Exp Med.

[6] Elsaesser H, Sauer K, Brooks DG (2009). IL-21 is required to control chronic viral infection. Science.

[7] Mehta DS, Wurster AL, Whitters MJ, Young DA, Collins M, Grusby MJ (2003). IL-21 induces the apoptosis of resting and activated primary B cells. J Immunol.

[8] He J, Tsai LM, Leong YA, Hu X, Ma CS, Chevalier N, Sun X, Vandenberg K, Rockman S, Ding Y (2013). Circulating precursor CCR7(lo)PD-1(hi) CXCR5(+) CD4(+) T cells indicate Tfh cell activity and promote antibody responses upon antigen reexposure. Immunity.

[9] Laichalk LL, Thorley-Lawson DA (2005). Terminal differentiation into plasma cells initiates the replicative cycle of Epstein-Barr virus in vivo. J Virol.

[10] Cohen JI, Jaffe ES, Dale JK, Pittaluga S, Heslop HE, Rooney CM, Gottschalk S, Bollard CM, Rao VK, Marques A (2011). Characterization and treatment of chronic active Epstein-Barr virus disease: a 28-year experience in the United States. Blood.

[11] Kim WY, Montes-Mojarro IA, Fend F, Quintanilla-Martinez L (2019). Epstein-Barr virus-associated T and NK-cell Lymphoproliferative diseases. Front Pediatr.

[12] Hislop AD, Taylor GS, Sauce D, Rickinson AB (2007). Cellular responses to viral infection in humans: lessons from Epstein-Barr virus. Annu Rev Immunol.

[13] Abe K, Takahashi A, Imaizumi H, Hayashi M, Okai K, Kanno Y, Watanabe H, Ohira H (2016). Interleukin-21 plays a critical role in the pathogenesis and severity of type I autoimmune hepatitis. Springerplus.

[14] Li Y, Tang L, Hou J (2015). Role of interleukin-21 in HBV infection: friend or foe?. Cell Mol Immunol.

[15] Hanna S, Etzioni A (2014). MHC class I and II deficiencies. J Allergy Clin Immunol.

[16] Pender MP: CD8+ T-cell deficiency, Epstein-Barr virus infection, vitamin D deficiency, and steps to autoimmunity: a unifying hypothesis. Autoimmune Dis 2012, 2012:189096. 10.1155/2012/189096 PMC3270541 22312480

[17] Tian Y, Cox MA, Kahan SM, Ingram JT, Bakshi RK, Zajac AJ (2016). A context-dependent role for IL-21 in modulating the differentiation, distribution, and abundance of effector and memory CD8 T cell subsets. J Immunol.

[18] Allard EL, Hardy MP, Leignadier J, Marquis M, Rooney J, Lehoux D, Labrecque N (2007). Overexpression of IL-21 promotes massive CD8+ memory T cell accumulation. Eur J Immunol.

[19] Nurieva RI, Chung Y, Hwang D, Yang XO, Kang HS, Ma L, Wang YH, Watowich SS, Jetten AM, Tian Q (2008). Generation of T follicular helper cells is mediated by interleukin-21 but independent of T helper 1, 2, or 17 cell lineages. Immunity.

[20] De Silva NS, Klein U (2015). Dynamics of B cells in germinal centres. Nat Rev Immunol.

[21] Kwun J, Manook M, Page E, Burghuber C, Hong J, Knechtle SJ (2017). Crosstalk between T and B cells in the germinal center after transplantation. Transplantation.

[22] Spolski R, Leonard WJ (2014). Interleukin-21: a double-edged sword with therapeutic potential. Nat Rev Drug Discov.

[23] Diehl SA, Schmidlin H, Nagasawa M, van Haren SD, Kwakkenbos MJ, Yasuda E, Beaumont T, Scheeren FA, Spits H (2008). STAT3-mediated up-regulation of BLIMP1 is coordinated with BCL6 down-regulation to control human plasma cell differentiation. J Immunol.

[24] Johnston RJ, Poholek AC, DiToro D, Yusuf I, Eto D, Barnett B, Dent AL, Craft J, Crotty S (2009). Bcl6 and Blimp-1 are reciprocal and antagonistic regulators of T follicular helper cell differentiation. Science.

[25] Peluso I, Fantini MC, Fina D, Caruso R, Boirivant M, MacDonald TT, Pallone F, Monteleone G (2007). IL-21 counteracts the regulatory T cell-mediated suppression of human CD4+ T lymphocytes. J Immunol.

[26] McNally A, Hill GR, Sparwasser T, Thomas R, Steptoe RJ (2011). CD4+CD25+ regulatory T cells control CD8+ T-cell effector differentiation by modulating IL-2 homeostasis. Proc Natl Acad Sci U S A.

[27] Fletcher LB, Veenstra RN, Loo EY, Hwang AE, Siddiqi IN, Visser L, Hepkema BG, Nolte IM, van den Berg A, Cozen W (2017). HLA expression and HLA type associations in relation to EBV status in Hispanic Hodgkin lymphoma patients. PLoS One.

